# Combination of RNA-Seq transcriptomics and iTRAQ proteomics reveal the mechanism involved in fresh-cut yam yellowing

**DOI:** 10.1038/s41598-021-87423-4

**Published:** 2021-04-08

**Authors:** Shuang Guo, Dan Wang, Yue Ma, Yan Zhang, Xiaoyan Zhao

**Affiliations:** 1 College of Food Science, Shenyang Agricultural University, Shenyang, 110866 Liaoning China; 2 Beijing Vegetable Research Center, Beijing Academy of Agriculture and Forestry Sciences, Beijing Key Laboratory of Agricultural Products of Fruits and Vegetables Preservation and Processing, Key Laboratory of Vegetable Postharvest Processing, Ministry of Agriculture and Rural Affairs, Beijing, 100097 China; 3 Longda Food Group Co. LTD, Shandong, 265231 China

**Keywords:** Biochemistry, Physiology

## Abstract

The aim of this study was to examine the regulation of transcriptomics and proteomics related to the yellowing of fresh-cut yams after storage. The comparison of yellow fresh-cut yam (YFY) vs. white fresh-cut yam (control) revealed 6894 upregulated and 6800 downregulated differentially expressed genes along with 1277 upregulated and 677 downregulated differentially expressed proteins. The results showed that the total carotenoids, flavonoids, and bisdemethoxycurcumin in YFY were higher than in the control due to the significant up-regulation of critical genes in the carotenoid biosynthesis pathway, flavonoid biosynthesis pathway, and stilbenoid, diarylheptanoid, and gingerol biosynthesis pathway. In addition, the tricarboxylic acid cycle and phenylpropanoid biosynthesis were both enhanced in YFY compared to the control, providing energy and precursors for the formation of yellow pigments. The results suggest that the synthesis of yellow pigments is regulated by critical genes, which might explain the yellowing of fresh-cut yam after storage.

## Introduction

Chinese yam (*Dioscorea opposita* Thunb.) is a well-known vegetable with high nutritional and medicinal values^1^, which contains mainly proteins, sugars, vitamins, fats, choline, amylase, iodine, iron, calcium, phosphorus, and other trace elements that are indispensable to the human body. In addition, the yam possesses may pharmacological effects including antioxidant, anti-aging, anti-tumor, and hypoglycemic. The yam also enhances immunity and is thus widely used in functional foods, health care products, medicine, and other industries^2,3^.

Fresh-cut fruits and vegetables are minimally processed, cold transported and ready-to-eat products, providing convenience and freshness to consumers^4^. Some consumers are allergic to the epiderm of Chinese yam^5^. Besides with the convenient eating characteristics, processing of fresh-cut Chinese yam can provide a good way to solve the allergy issues. The change in the color of the yam is a common quality issue during the processing and storage of fresh-cut yams, with browning being a limiting factor. Our recent study revealed that fresh-cut yam turns yellow during storage, which is attributed to various yellow substances including bisdemethoxycurcumin and other unknown compounds^6,7^. This yellowing process is different from the browning process during storage, which results from the polyphenol oxidase-catalyzed oxidation of phenolic compounds to orthoquinones^8^. However, a variety of specific metabolic pathways may be related to yellowing, and few prior studies have investigated these pathways fully.

Modern genetic research has demonstrated that most genes exercise their functions through the regulation of particular proteins. It has been extensively shown that post-transcriptional processing determines the levels of steady-state proteins^9^. RNA sequencing (RNA-Seq) is a method for transcript quantification that allows the more precise measurement of transcript levels and their isoforms compared to other approaches. Thus, RNA-Seq has been widely and successfully applied in transcript profiling, annotation, and gene identification in various plant species^10–12^.

Likewise, proteomics is also gaining recognition as a reliable and reproducible high-throughput approach for understanding biological processes^13,14^. Isobaric tags for relative and absolute quantification (iTRAQ) is a second-generation, gel-free proteomics approach that can be used to accurately quantify protein levels.

To clarify the yellowing mechanism of fresh-cut yam, we investigated the differentially expressed pathways responsible for yellowing. To do so, we carried out transcriptomic and proteomic profiling to identify changes in the expression levels of critical genes and proteins. GO and KEGG enrichment analyses were conducted along with a functional analysis to elucidate the pathways involved in the regulation of genes/proteins responsible for yellowing. The results provide valuable information for the preservation of yams.

## Results

### Apparent color analysis of fresh-cut yam

**Figure 1:**
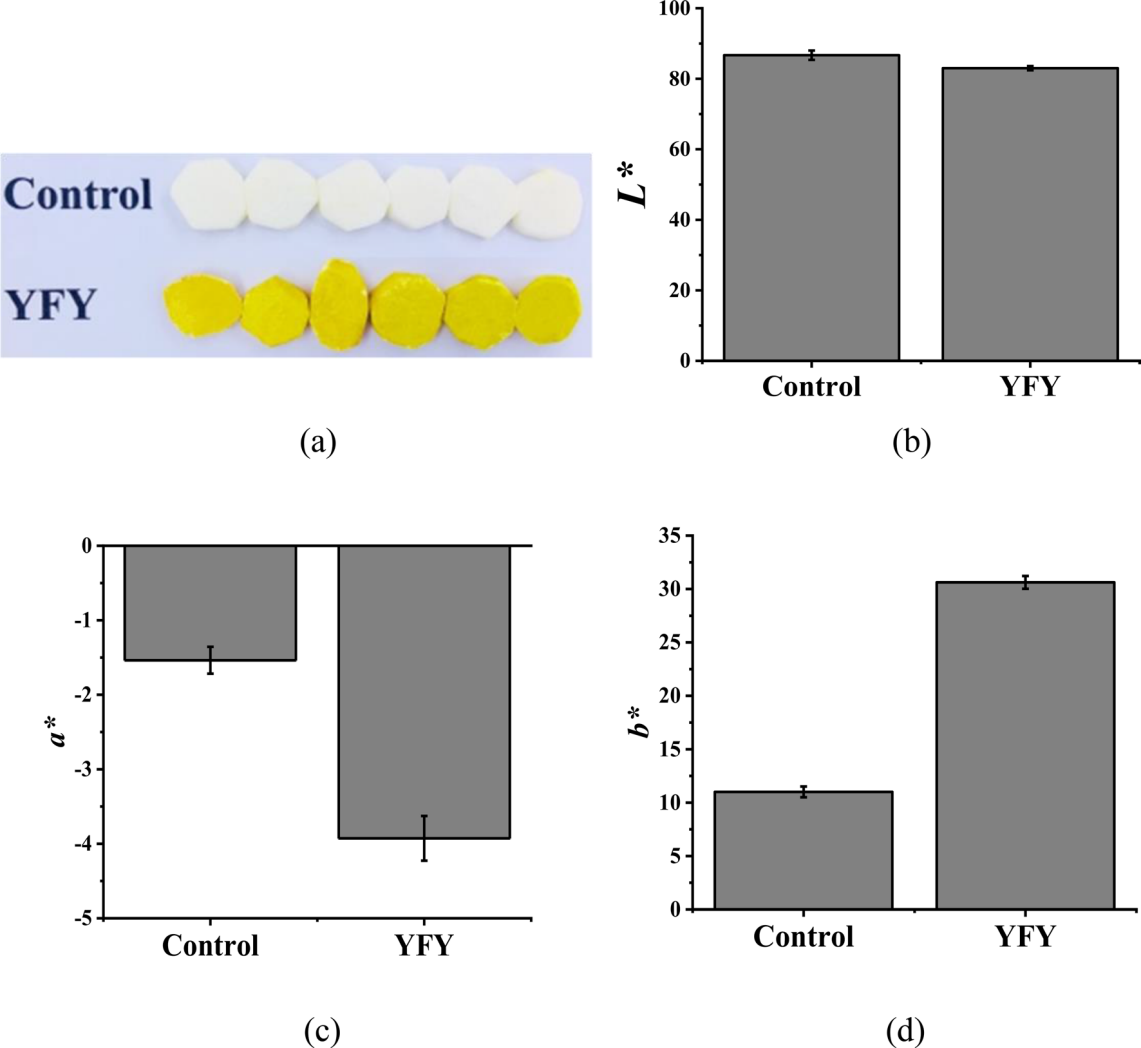
Apparent color analysis of fresh-cut yam: (**a**) yellowing of fresh-cut yam; and (**b**) L*, (**c**) a*, and (**d**) b* values of different groups. Each group included six replicate samples.

The color of fruits and vegetables is an important quality factor that reflects the freshness and indicates the market value of fresh products. In the present study, the color of fresh-cut yam became yellow after storage. The L*, a*, and b* values were evaluated, and the results for white fresh-cut yam (control) and YFY are shown in Fig. 1. As shown in Fig. 1, yams in the control group appeared white, which was the original color of the fresh-cut yam. In contrast, the yams in the YFY group appeared yellow, indicating significant yellowing during storage. Compared to the control, the YFY samples exhibited decreased L* and a* values but a significantly increased b* value, which is the most important index for evaluating yam yellowing, indicating that yellowing occurred during storage. The control and YFY samples were used for transcriptome and proteome analyses.

### Primary transcriptome analysis

By using statistical methods, we can make statistics and quality control of the tested sequence that directly reflect the quality of library construction and sequencing.

This method can also be used to analyze the base quality, base error rate, and base distribution of each sample. The total numbers of reads obtained for the two groups of fresh-cut yams are listed in Table 1. In the transcriptome analysis, we tested two RNA-Seq groups (control and YFY) with three biological replications, generating approximately 48,844,588 raw reads with sequencing error rates less than 3%. The raw reads were pre-processed by removing the reads containing adapter/primer sequences, reads with a percentage of indeterminate bases exceeding 10%, reads of low-quality sequences, and duplicates. A total of 48,301,943 clean reads were obtained for transcriptome assembly. Q20 is the percentage of bases number which is higher than 20 in the total bases number, and its ratio is 97.79%. Q30 is the percentage of bases number which is higher than 30 in the total bases number, and its ratio is 93.39%. Both Q20 and Q30 were higher than 90%. The GC content is the percentage of G and C bases out of the total number of bases; the GC content was approximately 50%. These results demonstrate that the sequencing level meets the quality needs.

**Table 1:** Sequencing statistics for control and YFY.

Sample	Raw reads	Raw bases	Clean reads	Clean bases	Error rate (%)	Q20 (%)	Q30 (%)	GC content (%)
Control1	47131910	7116918410	46602498	6938963552	0.0258	97.76	93.24	46.26
Control2	48427706	7312583606	47959846	7147194065	0.0252	98.01	93.84	46.36
Control3	43885330	6626684830	43482318	6468284270	0.0247	98.2	94.31	46.21
YFY1	45593078	6884554778	45098134	6702012579	0.0255	97.84	93.58	47.7
YFY2	61711348	9318413548	61018598	9065874144	0.0258	97.75	93.36	47.69
YFY3	46318156	6994041556	45650264	6781600407	0.0272	97.18	92.03	47.56
Average	48844588	7375532788	48301943	7183988170	0.0257	97.79	93.39	46.96

Supplementary Fig. S1a shows a correlation heatmap depicting the correlations between the three replicate samples based on the normalized expression results. The correlation coefficients between control samples were 96.3% (control 1 and control 2), 96.5% (control 1 and control 3), and 96.9% (control 2 and control 3). For the YFY samples, the correlation coefficients were 97.1% (YFY1 and YFY2), 97.0% (YFY1 and YFY3), and 97.5% (YFY3 and YFY2). The correlations indicate high sample repeatability. However, low correlation coefficients were obtained between the control and YFY, indicating that yellowing had a large effect on the samples. Principal component analysis (PCA) was used to explore the dataset and reveal hidden data patterns. After dimension reduction analysis, relative coordinate points were observed in the principal component plane. The distance between sample points represents the degree of similarity between the samples, with a shorter distance corresponding to greater similarity. In Supplementary Fig. S1b, the points of YFY samples are clustered together, indicating that YFY had the same characteristics. In contrast, the points of the control and YFY are scattered with respect to each other in Supplementary Fig. S1b with large separation distances, indicating that the control samples (control 1, control 2, and control 3) were significantly different from the YFY samples (YFY1, YFY2, and YFY3). More importantly, the process of yellowing greatly changed the characteristics of fresh-cut yam.

### Protein quality control

The high-resolution mass spectrometry results were statistically and qualitatively analyzed to reflect the quality of the detection results and data. A total of 866,811 spectra were produced from the iTRAQ experiments. By analyzing these spectra, we identified 175,705 known spectra, 45,580 peptides, 23,092 proteins, and 8459 protein groups. The spectrum utilization (i.e., the percentage of identified spectra out of the total spectra) was approximately 20% (Supplementary Fig. S2a), demonstrating that the chosen database was appropriate. The distribution of peptide length is shown in Supplementary Fig. S2b. Most peptides contained 5–20 amino acids, indicating that the enzymes had been sufficiently hydrolyzed. Supplementary Fig. S2c shows the distribution of protein mass. Proteins with masses of 1–21 kDa were the greatest in number (2251) followed by proteins with masses of 21–41 and 41–61 kDa. Peptide matching errors were within ± 20, and most of the errors were close to 0 (Supplementary Fig. S2d).

The results of the protein repetitive analysis are shown in Supplementary Fig. S2e,f. The coefficient of variation (CV) values for the control and YFY groups were both less than 0.1. The proportion of variation level between 10 and 20% accounted for the major part, demonstrating the high repetition in each group. The PCA results are shown in Supplementary Fig. S2g. Based on the score plot, the YFY samples were considerably different from the control samples; the YFY samples plotted on the negative side of the X axis, while the control samples plotted on the positive side. The results indicate that the YFY samples were quite different from the control samples, which can be attributed to the important effects of yellowing.

### Analysis of DEGs and DEPs

**Figure 2:**
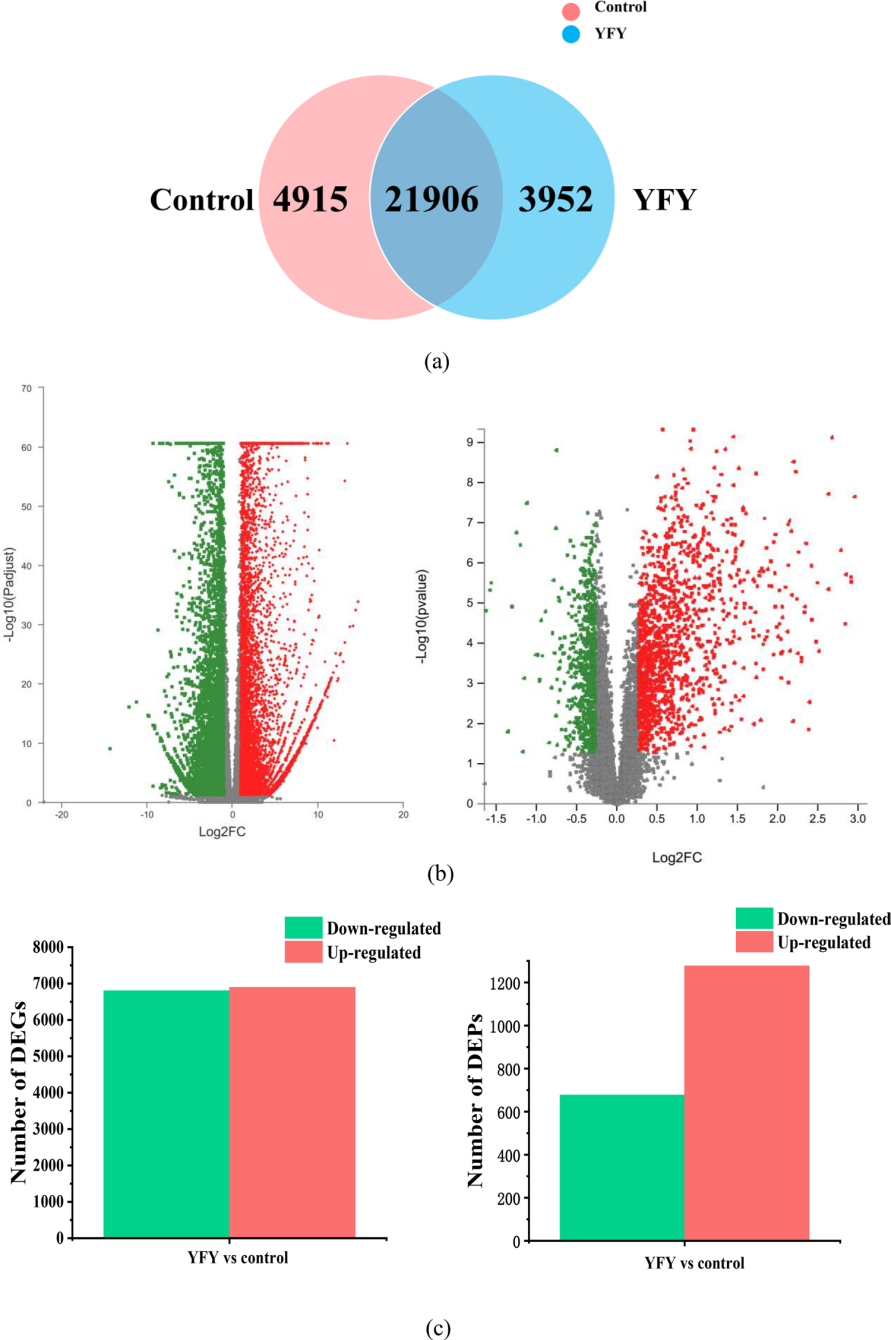
(**a**) Venn diagram in which the circles of different colors represent the number of UniGene/transcripts expressed in a group of samples. The areas of circle overlap represent the numbers of UniGene/transcripts shared by the groups. (**b**) Volcano plots of gene expression level and expressed protein abundance distribution. The green points represent downregulated genes/proteins, the red points represent upregulated genes/proteins, and the gray points represent non-regulated genes. (**c**) Numbers of DEGs and DEPs. The red bars denote upregulated genes/proteins, while the green bars show downregulated genes/proteins.

The software RNA-Seq by Expectation–Maximization (RESM) was used to analyze the expression levels of genes and transcripts using transcripts per million as a quantitative index. The number of transcripts was taken as the unit of calculation instead of the number of spliced segments. Factors such as the transcript length and the number of genes expressed in samples were considered comprehensively. A total of 21,906 transcripts were expressed in the control and YFY samples (Fig. 2a). DESeq2, a differential expression algorithm, was used for functional analysis to dig DEGs in different transcriptome groups. To generate resource of DEGs associated with the yellowing of fresh-cut yam, three independent biological replicates were produced for the transcriptome analysis of each group to determine the changes in gene expression for control and YFY. Transcripts found by DESeq2 with adjusted *P* values less than 0.05 were categorized as differentially expressed. Volcano plots were drawn to express the abundances and distributions of DEGs and DEPs. As shown in Fig. 2b, the distribution of DEGs and DEPs was in screening threshold dimensions. The abscissa was the multiple change value of DEGs/DEPs between two samples, which was obtained by dividing the expressions of the YFY and control samples. The ordinate was the *P* value of the DEGs/DEPs. A higher *P* value corresponds to a more significant differential expression. The abscissa and ordinate values are presented logarithmically in Fig. 2b. Each point in Fig. 2b represents a specific gene/protein; the red points represent significantly upregulated genes/proteins, the green points represent significantly downregulated genes/proteins, and gray points represent non-significantly different genes/proteins. After mapping all the genes, we could conclude that the green points represent downregulated expressions, while the red points are genes/proteins with upregulated expressions. Figure 2c shows the numbers of significantly up/downregulated genes and proteins. In the comparison of YFY vs the control, there were 6894 upregulated genes and 6800 downregulated genes. Among which, the expression of citrate synthase (TRINITY_DN6100_c0_g1) in YFY was up-regulated 45.3 times, phenylalanine ammonia lyase (TRINITY_DN24846_c0_g1) and chalcone synthase (TRINITY_DN15220_c2_g1) in YFY was up-regulated 67.4 and 37 times compared to control, which were critical regulators of the tricarboxylic acid cycle, phenylpropanoid biosynthesis pathway and flavonoids biosynthesis pathway. Meanwhile, the yellowing resulted in the increased expression of 1277 proteins and the decreased expression of 677 proteins. Such as, the protein expression of 5-*O*-(4-coumaroyl)-d-quinate 3′-monooxygenase (TRINITY_DN19359_c4_g1_i3_m.150690) in YFY was up-regulated 41.8 times. The expression of cinnamate 4-hydroxylase (TRINITY_DN20568_c1_g1_i13_m.285108) and 15-Cis-phytoene desaturase (TRINITY_DN18297_c0_g1_i10_m.312058) in YFY was up-regulated 19.7 and 31.2 times compared to control, which were critical regulators of the stilbenoid, diarylheptanoid, and gingerol biosynthesis pathway, phenylpropanoid biosynthesis pathway and carotenoid biosynthesis.

Thus, we can conclude that the expression of both genes and proteins are changed during storage, promoting the formation of yellow pigments in fresh-cut yam.

### GO and KEGG pathway enrichment analysis of DEGs

**Figure 3:**
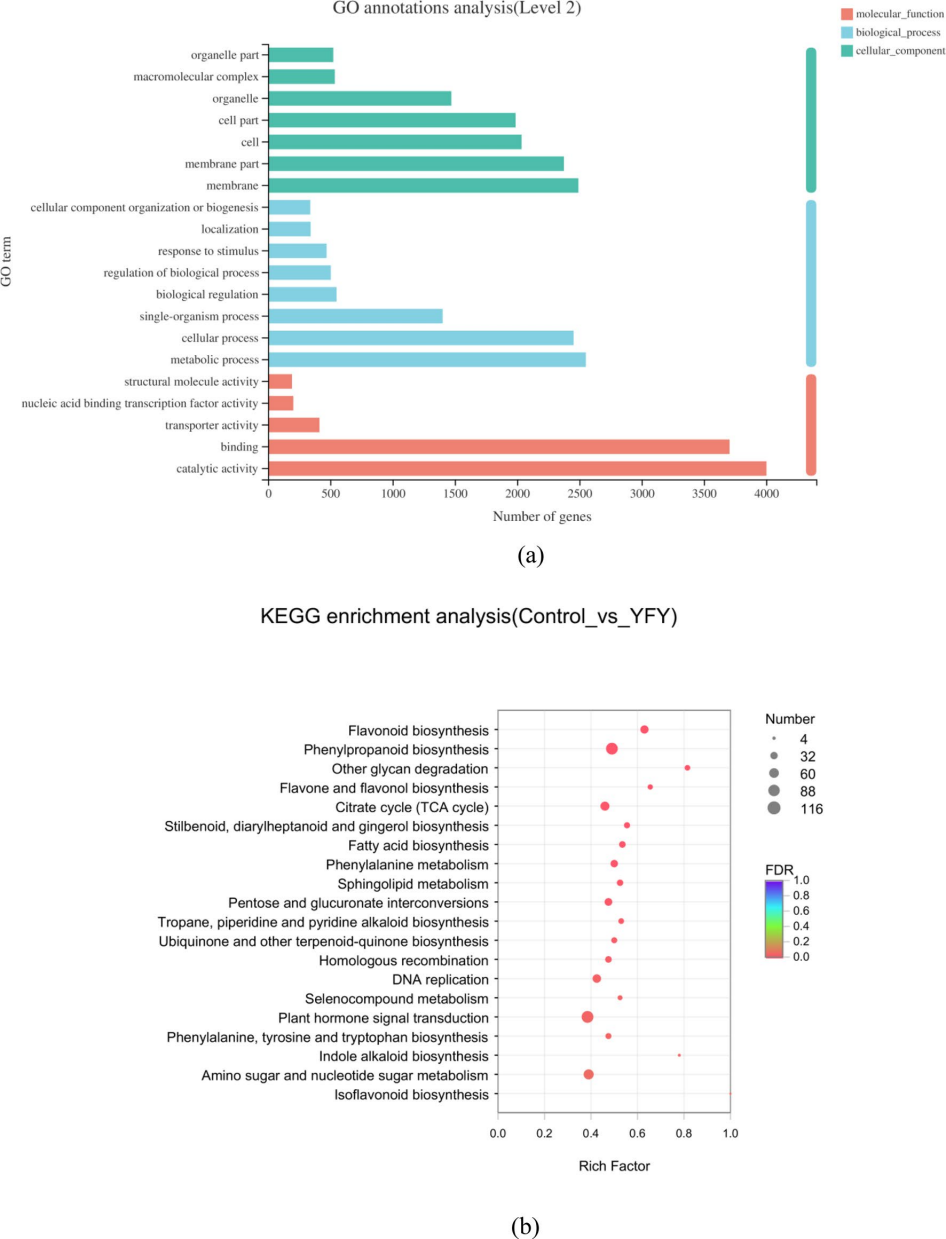
(**a**) GO terms enrichment of differentially expressed transcripts. The ordinate represents the secondary classification of GO, while the abscissa represents the number of genes included in the secondary classification. The color bars on the right represent the three branches of GO (blue, biological process; green, cellular component; dark pink: molecular function). (**b**) Statistics for the top 20 enriched pathway terms in the comparison of YFY vs control. The vertical axis represents the pathway name, and the abscissa represents the rich factor (i.e., the ratio of DEGs annotated in this pathway term to all gene numbers annotated in this pathway term; a greater rich factor indicates greater intensiveness). The size of the point indicates the number of genes in the pathway, while the color of the point corresponds to the Q value range.

We used Web Gene Ontology (WEGO) software for Gene Ontology (GO) functional classification to understand the distribution of genes at a macro level (Fig. 3a). According to the biological process, the DEGs that mapped to “cellular process” and “metabolic process” constituted a high proportion in yellowing fresh-cut yam, revealing high metabolic activity. This might reflect the biosynthesis and accumulation of bioactive secondary metabolites in yellowing fresh-cut yam. Two terms of “membrane” and “membrane part” occupied the highest part in cellular component category. Most of the DEGs were annotated as “binding” and “catalytic activity,” suggesting high catalytic activity in yellowing fresh-cut yam. Additionally, many genes were found to be related to “single-organism process” in the biological process area; “cell” and “cell part” in the cellular component; and “transporter activity” in the area of molecular function. In contrast, few genes were classified into the “cellular component organization or biogenesis,” “organelle part,” and “structural molecule activity” groups (Fig. 3a). Pathway enrichment analyses of the DEGs based on the Kyoto Encyclopedia of Genes and Genomes (KEGG) database were performed to study the gene interactions with each other during biological functions. Figure 3b shows a scatter plot for the top 20 KEGG enrichment results. Most genes were clustered in the phenylpropanoid biosynthesis and plant hormone signal transduction categories. The expressions of DEGs related to flavonoid biosynthesis and amino sugar and nucleotide sugar metabolism were higher in the YFY group compared to in the control group. Supplementary Table S1 shows the annotation pathways of DEGs in YFY vs. control. Of the total 63,357 annotated genes, 13,694 DEGs were produced, which enriched in 127 pathways, while 25 pathways were significant enriched. The main pathways (e.g., phenylpropanoid biosynthesis; flavonoid biosynthesis; and other glycan degradation pathways) affiliated with metabolism in the first category played an important role in the comparison of the YFY and control groups. These results demonstrate that different genes were produced and expressed, inducing the formation of yellow pigments in YFY.

### Overview of metabolism using MapMan

**Figure 4:**
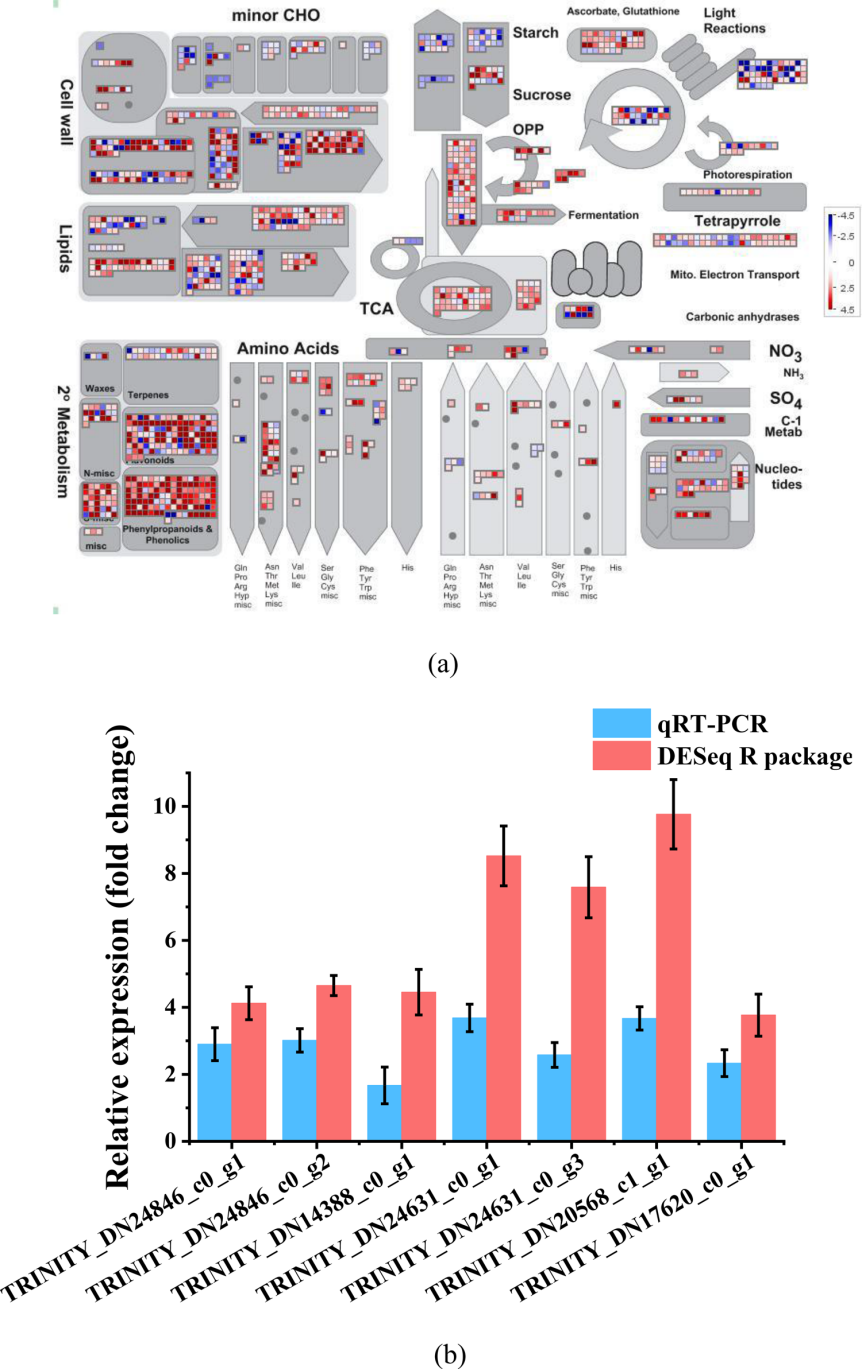
(**a**) Overview of DEGs involved in various metabolic processes under different treatments. Red represents upregulated genes, while blue represents downregulated genes. (**b**) Quantitative RT-PCR validation of gene expression.

Figure 4a shows a visual overview of metabolism obtained using MapMan. This figure shows the different functional categories that passed the cutoff (q value < 0.05 q and greater than two-fold change) for differential expression. In Fig. 4a, red represents upregulated genes, while blue represents downregulated genes. Compared to the control samples, the YFY samples exhibited higher expressions of genes related to ascorbate and glutathione metabolism, OPP, TCA and amino acids but lower expressions of genes related to light reaction and starch metabolism. The secondary metabolism contained numerous genes related to the synthesis of flavonoids, phenylpropanoids, and phenolics. The YFY group exhibited higher expressions of most genes related to flavonoids, phenylpropanoids, and phenolics. Genes in the cell wall and lipids showed different changes; some were upregulated, while some were downregulated. The results show that the yellowing of fresh-cut yam led to the accumulation of flavonoids, phenylpropanoids, and phenolics along with the degradation of starch and tetrapyrrole. The transformation of these substances may be an important reason for the yellowing.

### RT-PCR analysis

To further verify the results from the RNA-Seq work, seven transcripts of each sample were selected for quantitative RT-PCR analysis. The IDs of these genes are as follows: TRINITY_DN24846_c0_g2 (phenylalanine ammonia lyase), TRINITY_DN14388_c0_g1 (phenylalanine ammonia-lyase-like), TRINITY_DN24631_c0_g1 (4-coumarate-CoA ligase-like 1), TRINITY_DN24631_c0_g3 (4-hydroxycinnamoyl-CoA ligase 3), TRINITY_DN20568_c1_g1 (cytochrome P450 CYP73A100), TRINITY_DN17620_c0_g1 (trans-cinnamate 4-monooxygenase), and TRINITY_DN24846_c0_g1 (PAL5). These genes were found to be differentially expressed in the transcriptome of yellowing fresh-cut yam. The results of the RT-PCR analysis are shown in Fig. 4b. The trends in the expressions of all the unigenes from quantitative RT-PCR were consistent with the RNA sequencing analysis (Fig. 4b). These results demonstrate that the transcriptomic profiling data accurately reflected the response of fresh-cut yam to yellowing.

### GO and KEGG pathway enrichment analysis of DEPs

According to certain criteria (e.g., function, expression level, and expression differences), the protein list was obtained and analyzed. Some proteins related to the yellowing of fresh-cut yam were extracted, and their functions and expressions were studied.

**Figure 5:**
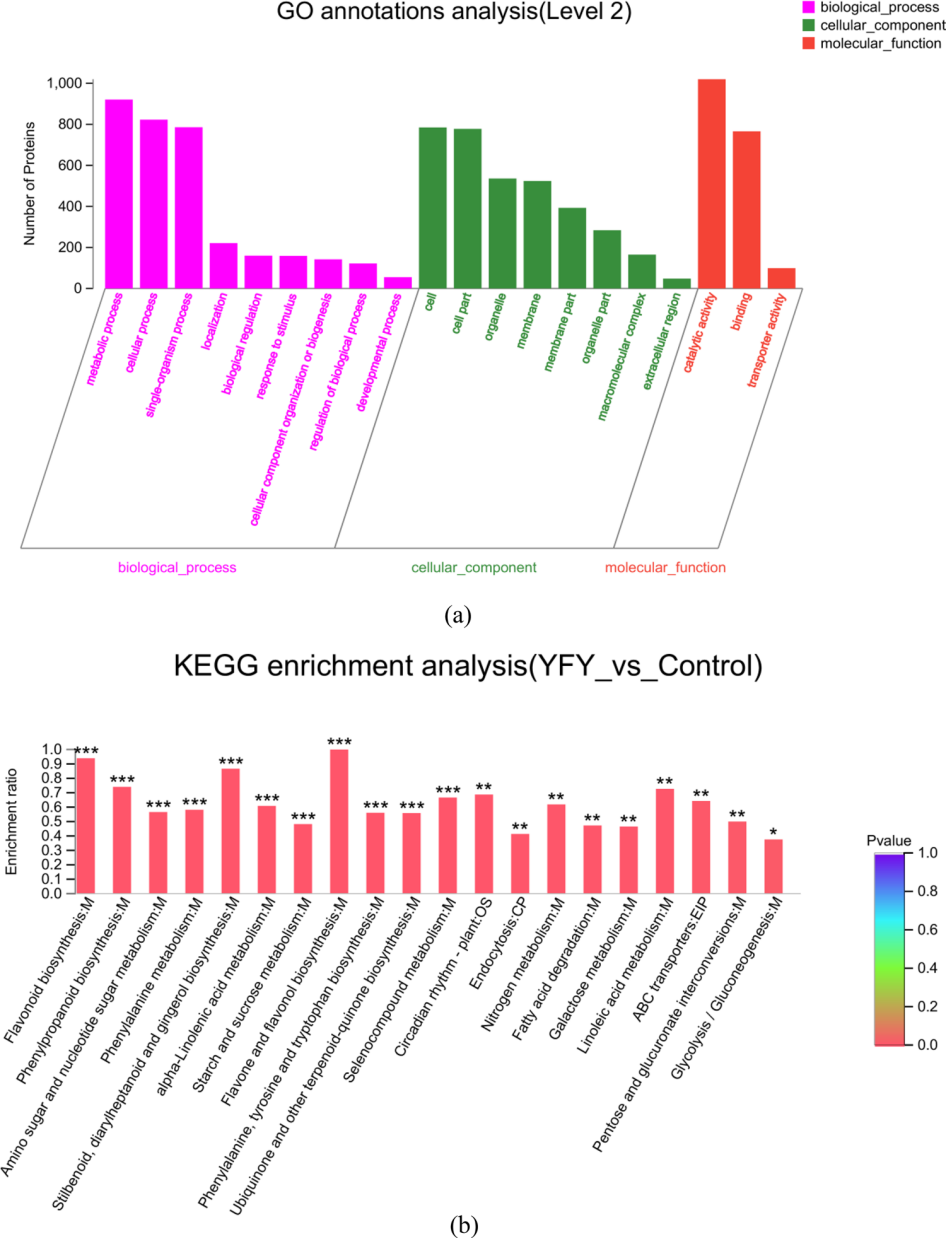
(**a**) GO terms enrichment of differentially expressed proteins. Each column represents a secondary classification of GO; a taller bar corresponds to more protein in the secondary classification. The abscissa represents the secondary GO classification term, and the ordinate represents the number of proteins annotated to each secondary classification. (**b**) KEGG enrichment analysis results. The abscissa represents the pathway name, and the ordinate represents the enrichment rate (i.e., ratio of the number of proteins enriched in the pathway to the number of proteins annotated in the pathway; a larger ratio corresponds to a greater degree of enrichment). The color gradient within the column indicates the significance of enrichment, with a darker color indicating greater enrichment in the KEGG term. ***Indicates P or FDR < 0.001; **indicates P or FDR < 0.01; and *indicates P or FDR < 0.05.

GO enrichment was used to analyze the key cellular process between the control and YFY samples. The distribution of proteins and their functional classifications are shown in Fig. 5a. Under the category of biological process, the majority of the DEPs were classified as “metabolic process” and “cellular process,” response to stimulus and regulation of biological process. The “cell,” “cell part,” and “organelle” categories were predominant within the cellular component category, and most genes were annotated in the “catalytic activity” and “binding categories. The comparative transcriptomic and proteomic data show that several cellular processes, including “metabolic process,” “cellular process,” and “catalytic activity” were affected by changes in key regulators at both the transcript and protein levels in our fresh-cut yam samples. Compared to the control samples (Fig. 5b), the YFY samples were enriched in most DEPs related to flavonoid biosynthesis; flavone and flavonol biosynthesis; phenylalanine biosynthesis; and stilbenoid, diarylheptanoid, and gingerol biosynthesis. These results reveal that different proteins were produced and expressed, and the enrichment in proteins involved in different pathways was basically consistent with the transcriptome results.

### Transcriptome and proteome correlation analysis

**Figure 6:**
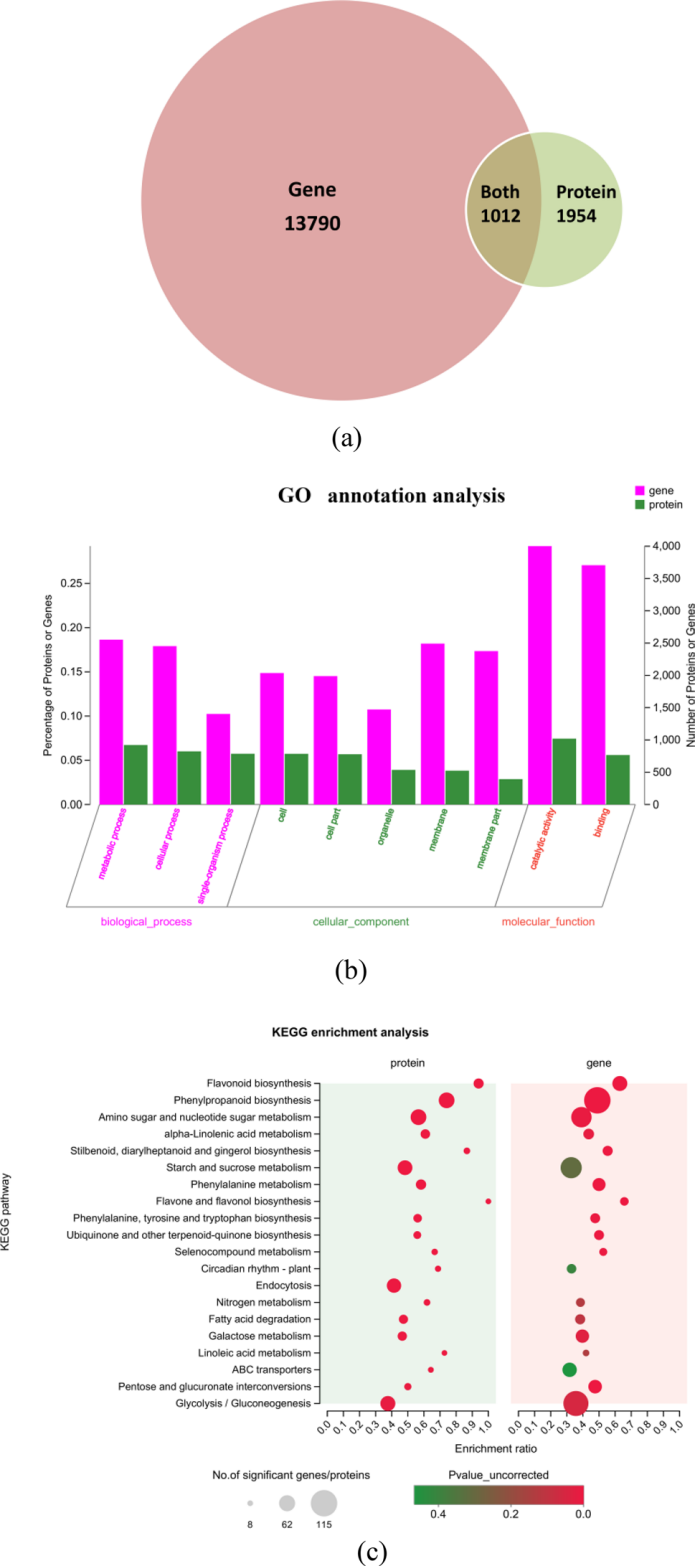
(**a**) Results of the correlation analysis of DEGs and DEPs. (**b**) Results of GO correlation analysis. The bars represent the secondary classifications of GO; bar height corresponds to the number of proteins or genes in the secondary classification; The abscissa represents the GO secondary classification term. The left ordinate represents the percentage of proteins or genes included in the secondary classification with respect to the total protein or gene number. The right ordinate represents the number of proteins or genes annotated to the secondary classification. (**c**) KEGG enrichment analysis results. The ordinate represents the pathway name, and the abscissa represents the enrichment rate (i.e., the ratio of the number of genes/proteins enriched in the pathway to the number of genes/proteins annotated in the pathway; a larger ratio corresponds to a greater degree of enrichment). Each bubble represents a path. The size of the bubble is directly proportional to the number of proteins or genes enriched in this pathway. The colors of the bubbles represent the p-values.

The Venn diagram in Fig. 6a presents the correlations in mRNA and protein differences among samples. The expression patterns of related molecules were studied at the transcription and protein levels. The analysis identified 13,790 DEGs and 1954 DEPs. Among which, 1012 genes were differentially expressed, and their corresponding translated proteins were also differentially expressed. Figure 6b shows the results of the GO correlation analysis, indicating that correlated proteins and genes were annotated to three biological processes: “metabolic process,” “cellular process,” and “single-organism process.” The correlated proteins and genes that mapped to “cell,” “cell part,” “organelle,” “membrane,” and “membrane part” constituted a high proportion of proteins and genes in the cellular component category. Furthermore, most of these correlated proteins and genes were annotated to the “binding” and “catalytic activity” molecular function categories. Indeed, as shown by the integrated comparative transcriptomic and proteomic data, several cellular processes were affected by changes in key regulators at both the transcript and protein levels in fresh-cut yam during yellowing, resulting in high metabolic activity, catalytic activity, and the accumulation of bioactive secondary metabolites in yellowing fresh-cut yam. KEGG enrichment of correlation data are shown in Fig. 6c. The correlated proteins and genes were both significantly annotated to flavonoid biosynthesis; phenylpropanoid biosynthesis; alpha-Linolenic acid metabolism; stilbenoid, diarylheptanoid, and gingerol biosynthesis; fatty acid degradation; ubiquinone and other terpenoid-quinone biosynthesis; phenylalanine metabolism; flavone and flavonol biosynthesis; and tyrosine metabolism. The *P* values of all these pathways were less than 0.1. The changes in these pathways might be critical factors in the yellowing of fresh-cut yam.

## Discussion

Vegetable color is generally accepted as one of the most important quality parameters and plays a decisive role in evaluating the quality of the vegetable product at the point of sale. In the present study, storage at 25 °C for 24 h decreased the L* and a* values but increased the b* value of fresh-cut yam, indicating yellowing (Fig. 1). According to our preliminary results, we extracted the yellow pigment in yellowing fresh-cut yam and identified its components. The yellow pigment was found to be composed primarily of bisdemethoxycurcumin, although other unknown substances were also present. To understand the yellowing mechanism of fresh-cut yam after storage at a molecular level, we compared the transcriptome and proteomic profiles of YFY and control samples. The important aspects of the physiological process related to yellowing are discussed below.

**Figure 7:**
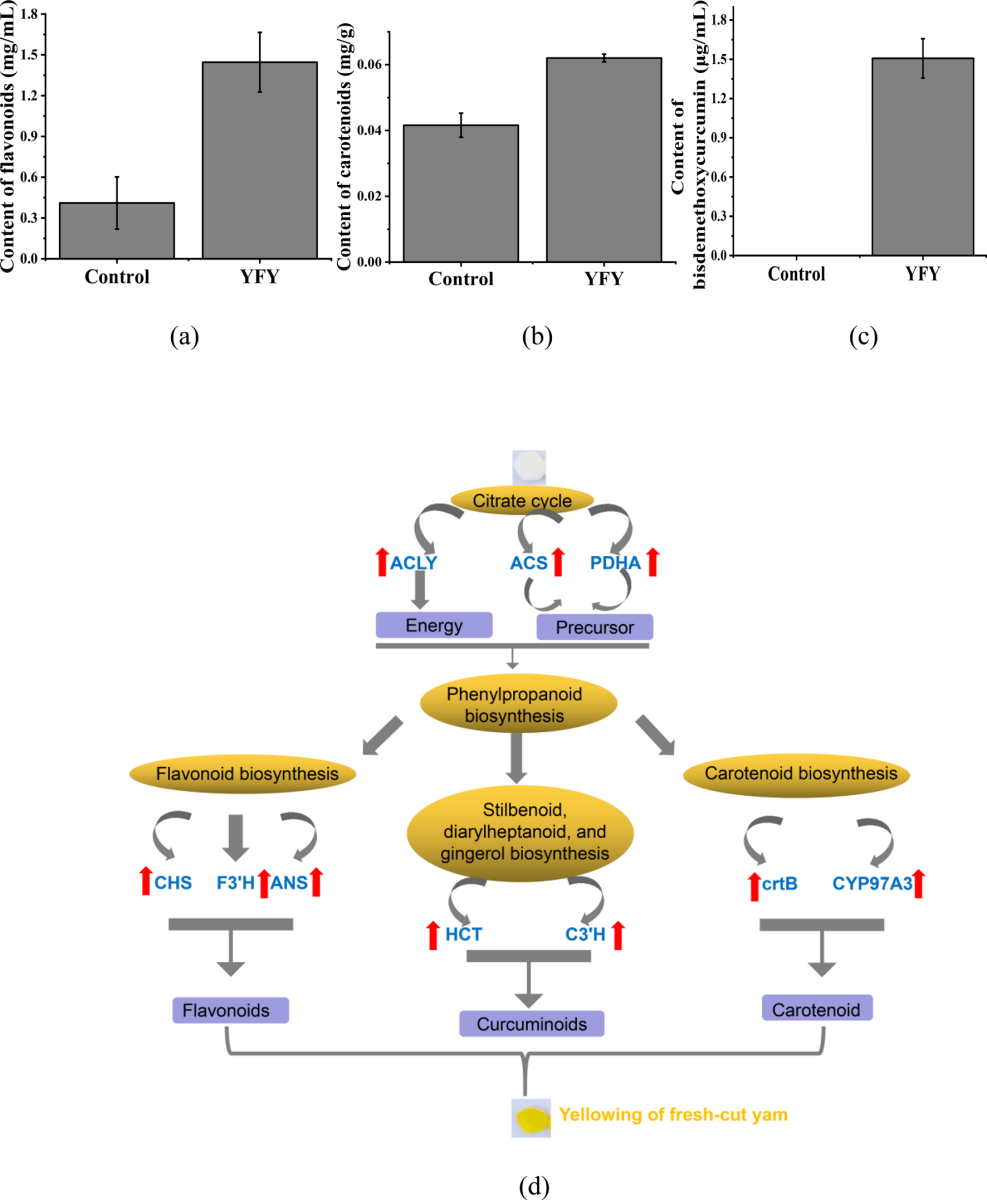
(**a**) Content of total flavonoids. (**b**) Content of total carotenoids. (**c**) Content of bisdemethoxycurcumin. (**d**) Hypothetical mechanism for the yellowing of fresh-cut yam.

The tricarboxylic acid cycle is the basic metabolic pathway shared by most organisms. The tricarboxylic acid cycle is the central hub of metabolism, participating in both catabolism and anabolism^15^. The tricarboxylic acid cycle also provides precursors for biosynthetic pathways^16^ such as the synthesis of acetyl-CoA, which has been implicated in the synthesis of secondary metabolites including flavonoids (Fig. 7a), stilbenoids, and isoprenoids. Acetyl-CoA is synthesized by two enzyme systems, the pyruvate dehydrogenase complex and acetyl-CoA synthetase systems^17^. In this study, pyruvate dehydrogenase complex (TRINITY_DN13442_c0_g1) and acetyl-CoA synthetase (TRINITY_DN19742_c0_g1) were respectively upregulated by 2.8 and 10.5 times in YFY compared to the control; the expressions of proteins TRINITY_DN15840_c2_g1_i7_m.43526 and TRINITY_DN13441_c0_g1_i1_m.148133 were also upregulated, resulting in the enhancement of acetyl-CoA metabolism. In addition, acetyl-CoA was carboxylated by acetyl-CoA carboxylase to form malonyl-CoA, the substrate for the biosynthesis of bisdemethoxycurcumin. The expression of acetyl-CoA carboxylase (TRINITY_DN9689_c0_g1) in YFY was 6.8 times higher than in the control. Therefore, the expressions of crucial genes related to acetyl-CoA biosynthesis and accumulation were significantly enhanced, providing precursors for the formation of yellow pigments (Fig. 7a–c). Furthermore, the tricarboxylic acid cycle can also meet most requirements for cell energy via the complete oxidation of acetyl-CoA. The activity of citrate synthase is fundamental to combine acetyl-CoA with oxaloacetate to form citrate^15^. In this study, the expressions of genes and proteins involved in citrate synthase (TRINITY_DN6100_c0_g1 and TRINITY_DN26760_c1_g2_i5_m.177726) were both significantly higher in YFY samples than in control samples. Therefore, the crucial genes/proteins involved in the oxidation of acetyl-CoA were upregulated, providing energy for the formation of yellow pigments.

The branches of the phenylpropanoid biosynthesis pathway give rise to thousands of compounds^18^. As the beginning of metabolic pathway of each branch (Fig. 7d), the phenylpropanoid biosynthesis pathway can provide precursors for the synthesis of yellow pigments. P-coumaroyl-CoA and cinnamoyl-CoA are the critical precursors of flavonoid biosynthesis and stilbenoid, diarylheptanoid, and gingerol biosynthesis^19,20^. The initial three steps in the pathway are catalyzed by phenylalanine ammonia lyase, cinnamate 4-hydroxylase, and 4-coumaroyl CoA-ligase, which are mandatory and provide the basis for all subsequent branches and resulting metabolites^21^. Phenylalanine ammonia lyase is one of the most well studied enzymes in the biosynthetic pathways of secondary metabolism. Phenylalanine ammonia lyase catalyzes the first step in the general phenylpropanoid pathway along with the deamination of phenylalanine to yield trans-cinnamic acid. Subsequently, cinnamate 4-hydroxylase and 4-coumarate CoA ligase catalyze the conversion of cinnamic acid to p-coumaroyl-CoA and 4-coumaroyl-CoA, respectively, which serve as intermediates and precursors for different phenylpropanoid compounds including flavonoids^22,23^ and curcuminoids. In this study, the expression levels of phenylalanine ammonia lyase (TRINITY_DN24846_c0_g1), cinnamate 4-hydroxylase (TRINITY_DN20568_c1_g1), and 4-coumaroyl CoA-ligase (TRINITY_DN19105_c0_g1) were significantly higher in YFY compared to the control. The DEGs can be correlated to the changes in the protein levels of phenylalanine ammonia lyase (TRINITY_DN27047_c0_g2_i5_m.279346), cinnamate 4-hydroxylase (TRINITY_DN20568_c1_g1_i13_m.285108), and 4-coumaroyl CoA-ligase (TRINITY_DN27047_c0_g2_i5_m.279346). This result indicates that phenylpropanoid biosynthesis was enhanced during storage, consistent with the high level of yellow pigments (Fig. 7a–c). The changes in the expressions of genes and proteins involved in phenylpropanoid biosynthesis were proportional to the degree of yellowing, further verifying that phenylpropanoid compounds play important roles in the yellowing of fresh-cut yam.

Flavonoids are a series of natural compounds that are widely distributed in plants and play important roles in diverse biological processes. For example, flavonoids impart various colors as they are major pigmentation factors^24^. In this study, the level of total flavonoids was analyzed in control and YFY samples (Fig. 7a). The YFY samples had a higher flavonoid content (0.22 mg/mL) than the control (0.19 mg/mL), indicating that total flavonoids increased during yellowing. Chalcone synthase, chalcone isomerase, and flavonoid 3′,5′-hydroxylase, are key enzymes in flavonoid biosynthesis and control the flux of flavonoids. In YFY, chalcone synthase (TRINITY_DN15220_c2_g1), chalcone isomerase (TRINITY_DN21557_c0_g1), and flavonoid 3′,5′-hydroxylase (TRINITY_DN15219_c0_g1) were respectively upregulated by 37, 25, and 16 times compared to the control, in agreement with the observed changes in protein levels. The expressions of chalcone synthase (TRINITY_DN15220_c2_g2_i4_m.227203) and chalcone isomerase (TRINITY_DN21557_c0_g1_i4_m.293684) in YFY were also upregulated by 4 and 7 times compared to the control, respectively. Flavonoid 3′-hydroxylase is also considered to be critical and rate-limiting enzyme. In this study, flavonoid 3′-hydroxylase (TRINITY_DN24846_c0_g1) was upregulated by 28 times in YFY compared to the control. The upregulated expressions of chalcone synthase, chalcone isomerase, flavonoid 3′,5′-hydroxylase, and flavonoid 3′-hydroxylase correspond directly to the increased content of total flavonoids in YFY compared to the control. Flavonoids such as eriodictyol and naringenin have been shown to lead to the yellowing of fresh-cut Chinese water chestnut^25,26^. However, the specific flavonoid substances responsible for the yellowing of fresh-cut yam merit further study.

The characteristic yellowish color of turmeric is attributed to the presence of polyphenolic pigment curcuminoids. The stilbenoid, diarylheptanoid, and gingerol biosynthesis pathway is responsible for the biosynthesis of curcumin, demethoxycurcumin, and bisdemethoxycurcumin, which has an intense yellow color. In the present study, bisdemethoxycurcumin was not detected in the control, while YFY contained 1.507 μg/mL bisdemethoxycurcumin (Fig. 7c), and its content was proportional to the degree of yellowing. This further verifies that bisdemethoxycurcumin plays an important role in the yellowing of fresh-cut yam. DEGs/DEPs were significantly enriched in the stilbenoid, diarylheptanoid, and gingerol biosynthesis pathway. Feruloyl-CoA, an intermediate of curcuminoids, was formed under the catalysis of shikimate *O*-hydroxycinnamoyltransferase, 5-*O*-(4-coumaroyl)-d-quinate, 3′-monooxygenase, and caffeoyl-CoA *O*-methyltransferase. In this study, the relative expressions of shikimate *O*-hydroxycinnamoyltransferase (TRINITY_DN22259_c0_g1, TRINITY_DN12166_c0_g3_i1_m.298158) and 5-*O*-(4-coumaroyl)-d-quinate 3′-monooxygenase (TRINITY_DN19359_c4_g1, TRINITY_DN19359_c4_g1_i3_m.150690) were upregulated in YFY. Moreover, the expression of caffeoyl-CoA *O*-methyltransferase (TRINITY_DN24830_c0_g2, TRINITY_DN21539_c3_g1_i3_m.294149) was approximately three and seven times higher in YFY than in the control. Curcumin synthase (TRINITY_DN8031_c0_g1) catalyzes formation of bisdemethoxycurcumin from p-coumaroyl-CoA, which was 1.8 times higher in YFY than in control. The synthesis of bisdemethoxycurcumin was enhanced in YFY compared to the control, indicating that these compounds were partly responsible for the yellowing of fresh-cut yam; this conclusion is supported by our recent results indicating that the yellow pigment was composed primarily of bisdemethoxycurcumin (73.7%)^6^.

Fruit color is related to the accumulation of carotenoids and other pigments, which play a major role in the yellow and orange pigmentation of maize kernel^27^. Due to the strong effect of carotenoids on fruit color, changes in the content of total carotenoids were analyzed in this study (Fig. 7b). The carotenoid content in the control (0.04 mg/g) was lower than that in YFY (0.06 mg/g), indicating that the content of total carotenoids increased during the yellowing of fresh-cut yam. Phytoene synthase, the first catalytic enzyme in carotenoid synthesis, was previously reported to control the synthesis of carotenoids in chromoplasts and determine the colors of mature pepper fruits^28^. In the present study, the expressions of phytoene synthase gene and protein (TRINITY_DN6245_c0_g1 and TRINITY_DN19316_c0_g1_i2_m.256713, respectively) eight and three times higher in YFY compared to the control, respectively, resulting in a significantly higher content of phytoene in YFY compared to control. Phytoene acts as a precursor to the formation of lutein, which is bright yellow. Lutein could be formed via the catalysis of α-carotene with beta-ring hydroxylase^29^. In this study, the expression of beta-ring hydroxylase (TRINITY_DN26312_c0_g1) in YFY was 12 times higher than in the control. Lycopene is the critical intermediate in the formation of lutein. 15-Cis-phytoene desaturase (TRINITY_DN18297_c0_g1, TRINITY_DN18297_c0_g1_i10_m.312058) and prolycopene isomerase (TRINITY_DN27028_c0_g1), which were upregulated in YFY compared to the control, catalyzed the formation of lycopene. These results suggest that the carotenoid biosynthesis pathway was enhanced in YFY, which might be critical for the accumulation of yellow substances.

According to the analysis above, in addition to bisdemethoxycurcumin and flavonoids, carotenoids might also contribute to the yellowing of fresh-cut yam. Based on the energy and precursors provided by the tricarboxylic acid cycle and phenylpropanoid biosynthesis, the yellow pigments were formed by the flavonoid biosynthesis, stilbenoid, diarylheptanoid, and gingerol biosynthesis, and carotenoid biosynthesis pathways (Fig. 7d).

## Materials and methods

### Samples

Fresh yam (*Dioscorea oppositifolia* L.), “Tiegun,” was purchased from Henan province, China. Uniformly sized yams without physical damage or disease were selected for experiment. The fresh yams were washed, peeled using a sharp, stainless-steel peeler, and chopped into small slices with thicknesses of 5 mm. The slices were then disinfected using 100 mL of NaClO solution (50 mg/L, pH 6.5) for 2 min. Gauze was used to wipe the water off the surface of each yam slice. The fresh-cut yam slices were packed in polyethylene plastic bags (70 ± 0.5 g) and preserved at 25 °C at 97% humidity for 24 h. The yams stored for 0 h were used as the control. After storage, the yam turned yellow (YFY). All materials were stored at − 80 °C until further analysis or used immediately.

### Color analysis

The external color of fresh-cut yam was measured using a chroma meter (Konica Minolta R-400 colorimeter; Minolta Co., Osaka, Japan), and measurements were taken on opposite sides of each slice. L* describes lightness (L* = 0 indicates black, L* = 100 indicates white), a* describes red–green intensity (a* > 0 indicates red, a* < 0 indicates green), and b*describes blue–yellow intensity (b* > 0 indicates yellow, b* < 0 indicates blue). The instrument was calibrated by a standard white and black board, in accordance with CIE specifications. 3 points were taken to be measured in each side of yam, and the mean for three pieces of fresh-cut yam in each replication.

### Analysis of total flavonoids in fresh-cut yam

The content of total flavonoids was investigated using a commercial kit (Solarbio, Beijing, China). Frozen tissue (0.5 g) was homogenized using a pestle and mortar in liquid nitrogen with 3 mL of extracting solution. The mixture was transferred to 10 mL centrifuge tube and extracted for 30 min. After centrifuging at 12,000 rpm for 10 min at 25 °C, the supernatant was collected as a source of flavonoids. Absorbance readings at 470 nm (UV-1800, Shimadzu, Japan) were taken and rutin was used for a standard curve. The content of total flavonoids in the supernatant was measured accurately using a detection kit for flavonoids in plants (Solarbio, Beijing, China) according to the manufacturer's instructions. Three biological repeats were applied in both YFY and Control.

### Analysis of total carotenoids in fresh-cut yam

The content of total carotenoids was investigated using a commercial kit (Solarbio, Beijing, China). Frozen tissue (0.5 g) was homogenized using a pestle and mortar in liquid nitrogen with 1 mL of distilled water and 20 mg reagent 1 under dark condition, and then transferred to a 10 mL centrifuge tube. The mixture was extracted for 3 h in the absence of light. During the process of extraction, the centrifuge tube was shaken twice to fully extract. After the tissue residue turning nearly white, which was in the bottom of tube, the mixture was centrifuged at 4000 rpm for 5 min. The supernatant was collected as a source of carotenoids. Absorbance readings at 440 nm (UV-1800, Shimadzu, Japan) were taken. The content of total carotenoids in the supernatant was measured accurately with a detection kit for carotenoids in plants (Solarbio, Beijing, China) according to the manufacturer's instructions. Three biological repeats were carried out in YFY and Control.

### Analysis of bisdemethoxycurcumin in fresh-cut yam

The content of bisdemethoxycurcumin was measured and quantified according to Zhao et al.^6^. Three biological repeats were carried out in both YFY and Control.

### RNA extraction

In the transcriptome analysis, three biological replicates of each treatment were used. Total RNA was extracted from the yam tissue using TRIzol Reagent according the manufacturer’s instructions (Invitrogen, Carlsbard, CA, USA), and genomic DNA was removed using DNase I (TaKara, Japan). The integrity and purity of the total RNA was then determined using a bioanalyzer (Agilent 2100 Bioanalyzer, Agilent Technologies, Inc., Santa Clara CA, USA) and quantified using an ND-2000 spectrophotometer (NanoDrop Thermo Scientific, Wilmington, DE, USA). Only high-quality RNA samples (OD260/280 = 1.8–2.2, OD260/230 ≥ 2.0, RIN ≥ 8.0, 28S:18S ≥ 1.0, > 2 μg) were used to construct the sequencing library.

### Library preparation and sequencing

Library preparation and sequencing were begun as described previously^30^.

### De novo assembly and annotation

The raw paired-end reads were trimmed and controlled for quality using the default parameters of SeqPrep (https://github.com/jstjohn/SeqPrep) and Sickle (https://github.com/najoshi/sickle). The clean data were then used for de novo assembly with Trinity (http://trinityrnaseq.sourceforge.net/)^31^. To retrieve the function annotations of the assembled transcripts, the transcripts were searched against the NCBI protein nonredundant (NR), string, and KEGG databases using BLASTX to identify the proteins with the highest sequence similarity to the given transcripts; typical cut-off E-values of less than 1.0 × 10^−5^ were used. The BLAST2GO (http://www.blast2go.com/b2ghome)^32^ program was used to obtain the GO annotations of unique assembled transcripts for describing biological processes, molecular functions, and cellular components. Metabolic pathway analysis was performed using the Kyoto Encyclopedia of Genes and Genomes (KEGG, http://www.genome.jp/kegg/).

### Differential expression analysis and functional enrichment

To identify differentially expressed genes (DEGs) between two different samples, the expression level of each transcript was calculated according to the fragments per kilobase of exon per million mapped reads (FRKM) method. RSEM (http://deweylab.biostat.wisc.edu/rsem/)^33^ was used to quantify gene and isoform abundances. Differential expression analysis was carried out in the R statistical package using EdgeR (Empirical analysis of Digital Gene Expression in R, http://www.bioconductor.org/packages/2.12.bioc/html/edgeR.html). GO and KEGG functional enrichment analyses were performed to identify which DEGs were significantly enriched in GO terms and metabolic pathways at Bonferroni-corrected P-value ≤ 0.05 compared to the whole-transcriptome background. GO functional enrichment and KEGG pathway analysis were carried out using Goatools (https://github.com/tanghaibao/Goatools) and KOBAS (http://kobas.cbi.pku.edu.cn/home.do), respectively^34^.

### RT-PCR analysis

To validate the transcriptomic data, seven DEGs were chosen for RT-PCR analysis according to the method of Zhang et al.^35^ with slight modifications. Total RNA was extracted with Trizol reagent (Invitrogen) from control and YFY plant tissues. RNA samples were treated with RNase-free DNase I (TaKaRa, Tokyo, Japan) to remove genomic DNA. RNA (10 µg) was used for reverse transcription with a PrimeScript RT reagent kit (Perfect Real Time, TaKaRa) according to the manufacturer’s instructions, and cDNA was used for RT-PCR analysis with specific primers. Quantitative RT-PCR was performed using an Applied Biosystems 7500 real-time PCR system with Power SYBR green chemistry (TaKaRa). Actin was quantified as an internal control and the 2^−ΔΔCt^ method was used to analyze the differential expression^36^. The mean of three biological replicates, for which three technical replicates were averaged, was presented. Genes were considered to be differentially expressed if (1) fold change ≥ 1.5 or ≤ 0.6 and (2) *P* value from post hoc ANOVA test ≤ 0.05.

### Total protein extraction

Five biological replicates of each treatment were used. The fresh-cut yam samples were resuspended in 1% crosslinked polyvinylpyrrolidone and BPP buffer (100 mM EDTA, 50 mM borax, 50 mM ascorbic acid, 30% sucrose, 100 mM TrisBase, 1% TritonX-100, 5 mM DTT, pH 8.0). Samples were treated using a high-throughput tissue grinder and vortexed three times for 40 s each time. After the samples were centrifugated at 12,000×*g* at 4 °C for 20 min, the protein supernatants were mixed with equal volumes of Tris-saturated phenol and vortexed for 10 min at 4 °C. After centrifugation at 12,000×*g* at 4 °C for 20 min, the phenol phase was added to an equal volume of BPP and vortexed for 10 min at 4 °C. After centrifugation at 12,000×*g* at 4 °C for 20 min, the phenol phase was added to a five-fold volume of pre-cooled methanolic ammonium acetate solution to precipitate protein for 12 h at − 20 °C. After centrifugation at 12,000×*g* at 4 °C for 20 min, the pellet was rinsed twice with 90% acetone and then dried under vacuum. The acetone-dried powder was resuspended in protein lysis buffer containing 8 M urea, 1% SDS, and an appropriate amount of protease inhibitor. The mixture was treated ultrasonically for 20 min, and after centrifugation at 12,000×*g* at 4 °C for 20 min, the concentration of protein was determined using the bicinchoninic acid (BCA) method with a BCA Protein Assay Kit (Thermo, MA, USA). Protein quantification was performed according to the kit protocol.

### Protein digestion and TMT labeling

Protein was digested with trypsin (Promega, Madison, WI, USA) and incubated at 37 °C with a mass ratio of 1:50 (enzyme/substrate) overnight. The iTRAQ labeling was performed according to the manufacturer’s illustration (Applied Biosystems, Sciex, Foster City, CA, USA). All labeled peptides were pooled, desalted, and vacuum-dried^37^.

### High-pH RPLC separation

The pooled samples were fractionated using an ultraperformance liquid chromatography (Acquity, Waters, USA) with an Acquity UPLC BEH C18 Column (1.7 µm, 2 mm × 150 mm; Waters, USA) to increase the proteomic depth. Briefly, the peptides were first separated via gradient elution (Phase A: 5 mM ammonium hydroxide solution containing 2% acetonitrile, pH 10; Phase B: 5 mM ammonium hydroxide solution containing 80% acetonitrile, pH 10) over 48 min at a flow rate of 200 μL/min with the following gradient: 0–5% buffer B for 2 min; 5% buffer B from 2–17 min; 5–30% buffer B from 17–35 min; 30%–36% buffer B from 35–38 min; 36–42% buffer B from 38–39 min; 42–100% buffer B from 39–40 min; 100–0% buffer B from 40–45 min; and 0% buffer B for 3 min. Thirty fractions were collected from each sample. These fractions were pooled, resulting in 15 total fractions per sample.

### Liquid chromatography tandem mass spectrometry (LC–MS/MS) analysis

The labeled peptides were analyzed by online nanoflow LC–MS/MS performed on an EASY-nLC system (Thermo, USA) connected to a Q Exactive quadrupole orbitrap mass spectrometer (Thermo, USA) using a nanoelectrospray ion source. Briefly, a C18 reversed-phase column (75 μm × 25 cm; Thermo, USA) was equilibrated with solvent A (A: 2% acetonitrile with 0.1% formic acid) and solvent B (B: 80% ACN with 0.1% formic acid). The peptides were eluted using the following gradient program: 0–1 min, 0–5% B; 1–63 min, 5–23% B; 63–77 min, 23–29% B; 77–86 min, 29–38% B; 86–88 min, 38–48% B; 88–89 min, 48–100% B; and 89–95 min, 100% B) at a flow rate of 300 nL/min. The spectrometer was operated in the data-dependent mode to automatically switch between full-scan MS and MS/MS acquisition. The full-scan survey (*m*/*z* 350–1300) was acquired using an Orbitrap with a resolution of 70,000. The dynamic exclusion time was set to 18 s. The 20 most intense MS peaks were sequentially isolated and fragmented in the octopole collision cell using high-energy collisional dissociation with a resolution of 35,000 in fast-scanning mode.

### Protein identification

With ProteomeDiscoverer (Thermo Scientific, Version 2.2), analysing raw data against transcriptome was conducted. The searching of MS/MS was according to the criteria: Two missed cleavages in the trypsin digesting were allowed, with a mass tolerance of 0.05 Da (10 ppm). Choosing TMT of the N-terminus and lysine side chains of peptidases and carbamido methylation of cysteine to fix modification. A false discovery rate (FDR) threshold of less than 1% were used.

## Supplementary Information

Supplementary Figures.

Supplementary Table 1.
