# Supplementary material for: Combination of RNA-Seq transcriptomics and iTRAQ proteomics reveal the mechanism involved in fresh-cut yam yellowing
Source: Sci Rep. 2021 Apr 8;11:7755. doi: 10.1038/s41598-021-87423-4 (PMC8032744; doi:10.1038/s41598-021-87423-4)
Supplement: Supplementary file 1 — Supplementary Figures. [file 41598_2021_87423_MOESM1_ESM.doc]

**Combination of RNA-Seq transcriptomics and iTRAQ proteomics reveal the mechanism involved in fresh-cut yam yellowing**

Shuang Guo ^a, b, §^, Dan Wang ^b, §^, Yue Ma ^b^, Yan Zhang ^c^, Xiaoyan Zhao ^b^, *

a College of Food Science, Shenyang Agricultural University, Shenyang, Liaoning 110866, China

b Beijing Vegetable Research Center, Beijing Academy of Agriculture and Forestry Sciences, Beijing Key Laboratory of Agricultural Products of Fruits and Vegetables Preservation and Processing, Key Laboratory of Vegetable Postharvest Processing, Ministry of Agriculture and rural affairs, Beijing 100097, China.

c Longda Food Group Co. LTD, Shandong 265231, China.

*Corresponding author. Tel.: +86-10-51503057, Fax: +86-10-51503053, E-mail: xiaoyanzhao001@163.com

§ These authors contributed equally to this work.


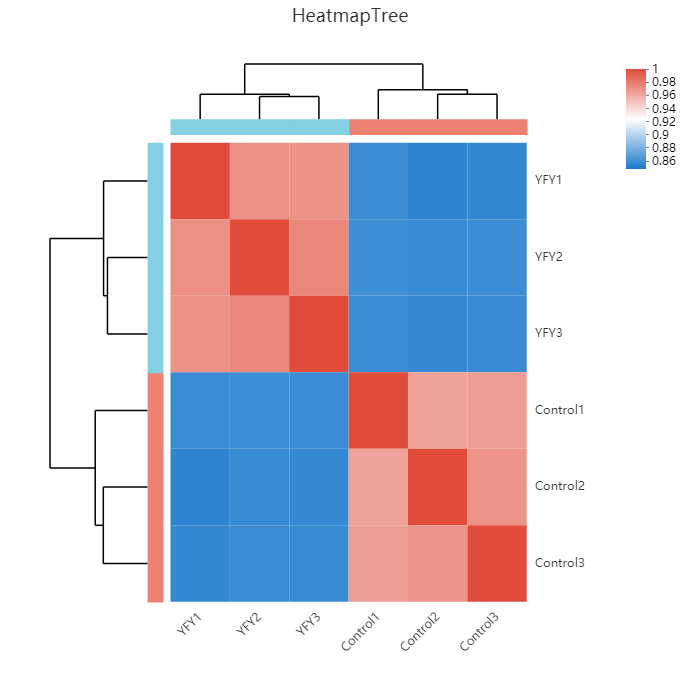


(a)


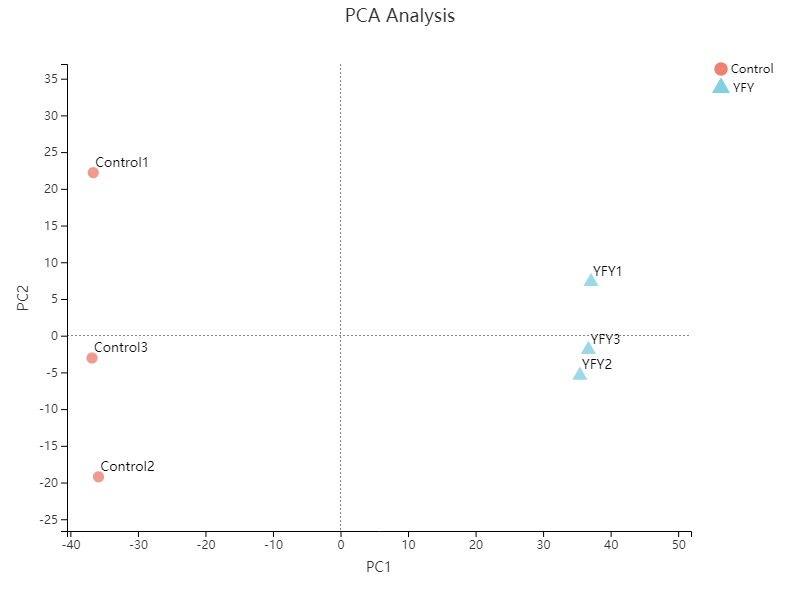


(b)

**Fig. S1** (a) Correlation heatmap of yam samples. The gradient color barcode at the top right indicates the maximum value in red and the minimum in blue. Each grid represents the correlation between two samples, and different colors represent the correlation coefficients between samples. (b) PCA analysis results based on the transcriptome information. The distance between sample points represents the degree of similarity between the samples, with a shorter distance corresponding to greater similarity.


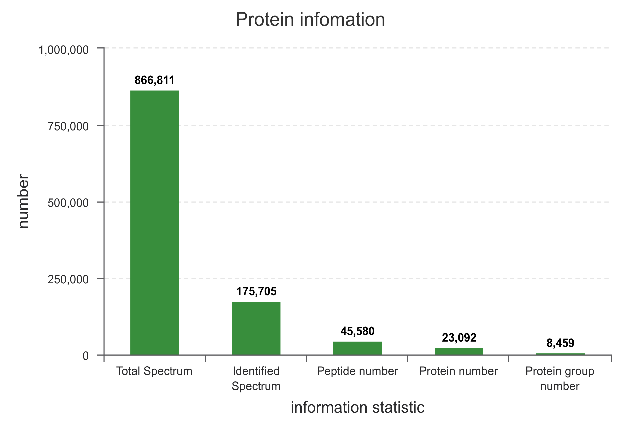

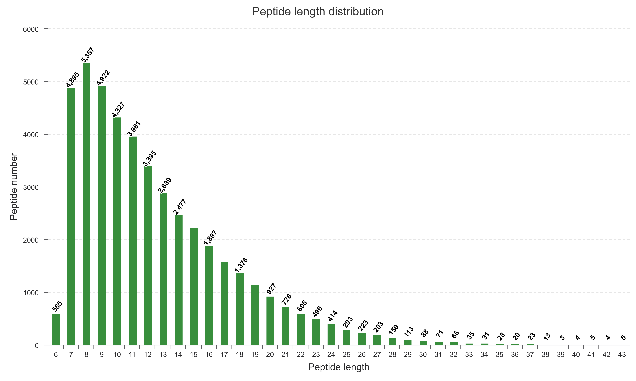


(a) (b)


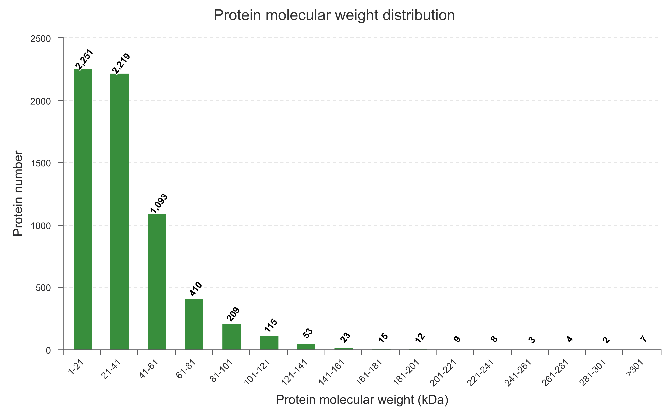
**
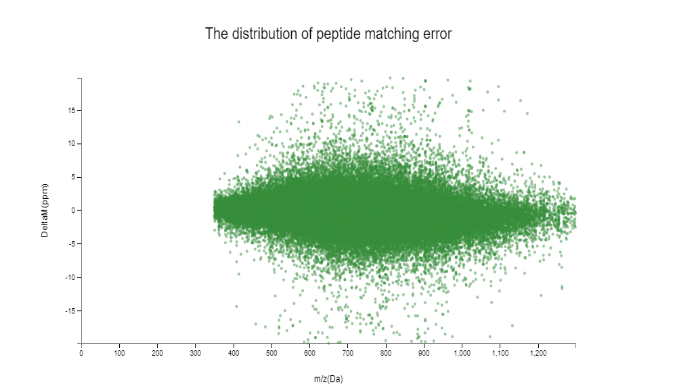
**

(c) (d)


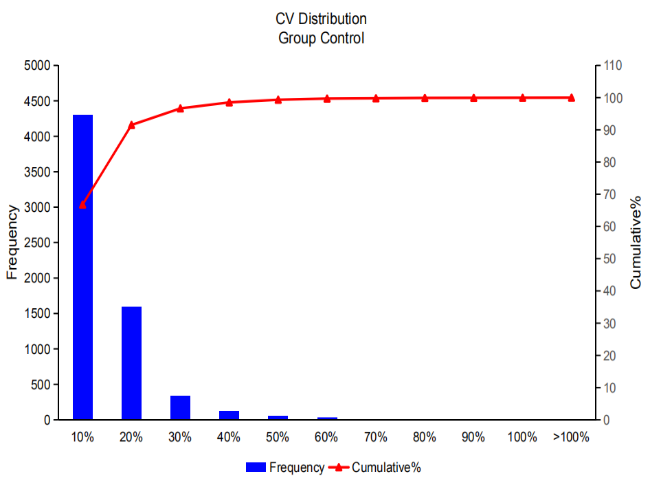

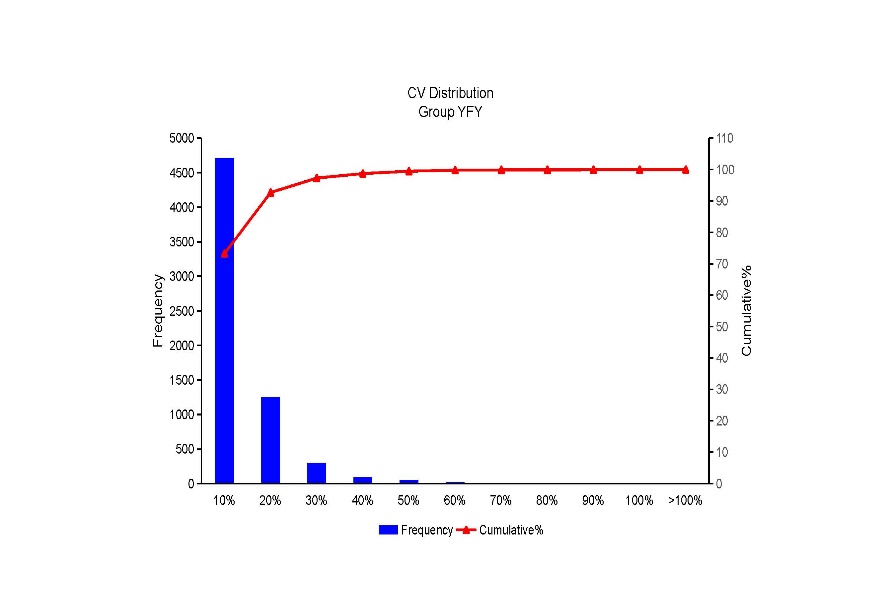


(e) (f)


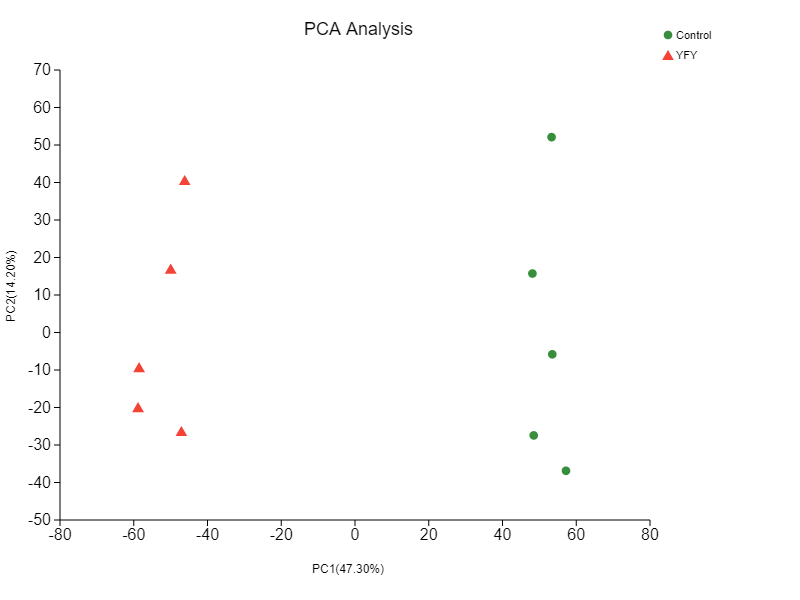


(g)

**Fig. S2** Identification and analysis of the proteome: (a) protein information; (b) peptide length distribution; (c) protein molecular weight distribution; and (d) distribution of peptide matching error. (e) Plots of the control group showing the CV values (%) for comparing protein quantifications over the lengths of identified peptides. (f) Plots of the YFY group showing the CV values (%) to compare protein quantifications over the lengths of identified peptides. (g) PCA analysis results of samples according to the proteomic information. The distance between sample points represents the degree of similarity between the samples, with a shorter distance corresponding to greater similarity.
