# Supplementary material for: Combination of RNA-Seq transcriptomics and iTRAQ proteomics reveal the mechanism involved in fresh-cut yam yellowing
Source: Sci Rep. 2021 Apr 8;11:7755. doi: 10.1038/s41598-021-87423-4 (PMC8032744; doi:10.1038/s41598-021-87423-4)
Supplement: Supplementary file 2 — Supplementary Table 1. [file 41598_2021_87423_MOESM2_ESM.doc]

**Combination of RNA-Seq transcriptomics and iTRAQ proteomics reveal the mechanism involved in fresh-cut yam yellowing**

Shuang Guo ^a, b, §^, Dan Wang ^b, §^, Yue Ma ^b^, Yan Zhang ^c^, Xiaoyan Zhao ^b^, *

a College of Food Science, Shenyang Agricultural University, Shenyang, Liaoning 110866, China

b Beijing Vegetable Research Center, Beijing Academy of Agriculture and Forestry Sciences, Beijing Key Laboratory of Agricultural Products of Fruits and Vegetables Preservation and Processing, Key Laboratory of Vegetable Postharvest Processing, Ministry of Agriculture and rural affairs, Beijing 100097, China.

c Longda Food Group Co. LTD, Shandong 265231, China.

*Corresponding author. Tel.: +86-10-51503057, Fax: +86-10-51503053, E-mail: xiaoyanzhao001@163.com

§ These authors contributed equally to this work.

Table S1 Annotation pathways of DEGs in YFY vs. Control

| Num | Pathway id | | Description | Ratio in study | | Ratio in pop | P value corrected | First Category | Second Category |
| --- | --- | --- | --- | --- | --- | --- | --- | --- | --- |
| 56 | map00941 | Flavonoid biosynthesis | | | 56/4281 | 89/13729 | 8.03538E-08 | Metabolism | Biosynthesis of other secondary metabolites |
| 115 | map00940 | Phenylpropanoid biosynthesis | | | 115/4281 | 235/13729 | 3.33614E-07 | Metabolism | Biosynthesis of other secondary metabolites |
| 26 | map00511 | Other glycan degradation | | | 26/4281 | 32/13729 | 4.51397E-07 | Metabolism | Glycan biosynthesis and metabolism |
| 23 | map00944 | Flavone and flavonol biosynthesis | | | 23/4281 | 35/13729 | 0.000856101 | Metabolism | Biosynthesis of other secondary metabolites |
| 67 | map00020 | Citrate cycle (TCA cycle) | | | 67/4281 | 146/13729 | 0.002304158 | Metabolism | Carbohydrate metabolism |
| 31 | map00945 | Stilbenoid, diarylheptanoid and gingerol biosynthesis | | | 31/4281 | 56/13729 | 0.002339178 | Metabolism | Biosynthesis of other secondary metabolites |
| 36 | map00061 | Fatty acid biosynthesis | | | 36/4281 | 67/13729 | 0.002587994 | Metabolism | Lipid metabolism |
| 46 | map00360 | Phenylalanine metabolism | | | 46/4281 | 92/13729 | 0.002602313 | Metabolism | Amino acid metabolism |
| 34 | map00600 | Sphingolipid metabolism | | | 34/4281 | 65/13729 | 0.004377948 | Metabolism | Lipid metabolism |
| 49 | map00040 | Pentose and glucuronate interconversions | | | 49/4281 | 103/13729 | 0.004437185 | Metabolism | Carbohydrate metabolism |
| 27 | map00960 | Tropane, piperidine and pyridine alkaloid biosynthesis | | | 27/4281 | 51/13729 | 0.011406758 | Metabolism | Biosynthesis of other secondary metabolites |
| 30 | map00130 | Ubiquinone and other terpenoid-quinone biosynthesis | | | 30/4281 | 60/13729 | 0.018751246 | Metabolism | Metabolism of cofactors and vitamins |
| 36 | map03440 | Homologous recombination | | | 36/4281 | 76/13729 | 0.021687505 | Genetic Information Processing | Replication and repair |
| 60 | map03030 | DNA replication | | | 60/4281 | 142/13729 | 0.030355249 | Genetic Information Processing | Replication and repair |
| 20 | map00450 | Selenocompound metabolism | | | 20/4281 | 38/13729 | 0.03555706 | Metabolism | Metabolism of other amino acids |
| 116 | map04075 | Plant hormone signal transduction | | | 116/4281 | 303/13729 | 0.03731373 | Environmental Information Processing | Signal transduction |
| 30 | map00400 | Phenylalanine, tyrosine and tryptophan biosynthesis | | | 30/4281 | 63/13729 | 0.038341256 | Metabolism | Amino acid metabolism |
| 7 | map00901 | Indole alkaloid biosynthesis | | | 7/4281 | 9/13729 | 0.038484822 | Metabolism | Biosynthesis of other secondary metabolites |
| 84 | map00520 | Amino sugar and nucleotide sugar metabolism | | | 84/4281 | 215/13729 | 0.054184333 | Metabolism | Carbohydrate metabolism |
| 4 | map00943 | Isoflavonoid biosynthesis | | | 4/4281 | 4/13729 | 0.059976343 | Metabolism | Biosynthesis of other secondary metabolites |
| 32 | map00350 | Tyrosine metabolism | | | 32/4281 | 72/13729 | 0.07291021 | Metabolism | Amino acid metabolism |
| 34 | map00592 | alpha-Linolenic acid metabolism | | | 34/4281 | 78/13729 | 0.07649766 | Metabolism | Lipid metabolism |
| 6 | map00604 | Glycosphingolipid biosynthesis - ganglio series | | | 6/4281 | 8/13729 | 0.079855647 | Metabolism | Glycan biosynthesis and metabolism |
| 75 | map00620 | Pyruvate metabolism | | | 75/4281 | 195/13729 | 0.090023166 | Metabolism | Carbohydrate metabolism |
| 58 | map00564 | Glycerophospholipid metabolism | | | 58/4281 | 146/13729 | 0.090625012 | Metabolism | Lipid metabolism |

Ratio in study: the proportion of KEGG in the target gene set. The numerator is the number of unigene/transcript enriched in KEGG, and the denominator is the total number of unigene/transcript with KEGG annotation in the gene set; Ratio in Pop: the proportion of KEGG in the background unigene/transcript (all unigene/transcript obtained by sequencing). The numerator is the number of all background unigene/transcript enriched in the KEGG, and the denominator is the total number of background unigene/transcript with KEGG annotation in the gene set.
